# Compass Cells in the Brain of an Insect Are Sensitive to Novel Events in the Visual World

**DOI:** 10.1371/journal.pone.0144501

**Published:** 2015-12-04

**Authors:** Tobias Bockhorst, Uwe Homberg

**Affiliations:** Animal Physiology, Faculty of Biology, Philipps Universität Marburg, Marburg, Germany; University of Arizona, UNITED STATES

## Abstract

The central complex of the insect brain comprises a group of neuropils involved in spatial orientation and memory. In fruit flies it mediates place learning based on visual landmarks and houses neurons that encode the orientation for goal-directed locomotion, based on landmarks and self-motion cues for angular path-integration. In desert locusts, the central complex holds a compass-like representation of head directions, based on the polarization pattern of skylight. Through intracellular recordings from immobilized locusts, we investigated whether sky compass neurons of the central complex also represent the position or any salient feature of possible landmarks, in analogy to the observations in flies. Neurons showed strongest responses to the novel appearance of a small moving square, but we found no evidence for a topographic representation of object positions. Responses to an individual square were independent of direction of motion and trajectory, but showed rapid adaptation to successive stimulation, unaffected by changing the direction of motion. Responses reappeared, however, if the moving object changed its trajectory or if it suddenly reversed moving direction against the movement of similar objects that make up a coherent background-flow as induced by ego-motion. Response amplitudes co-varied with the precedent state of dynamic background activity, a phenomenon that has been related to attention-dependent saliency coding in neurons of the mammalian primary visual cortex. The data show that neurons of the central complex of the locust brain are visually bimodal, signaling sky compass direction and the novelty character of moving objects. These response properties might serve to attune compass-aided locomotor control to unexpected events in the environment. The difference to data obtained in fruit flies might relate to differences in the lifestyle of landmark learners (fly) and compass navigators (locust), point to the existence of parallel networks for the two orientation strategies, or reflect differences in experimental conditions.

## Introduction

Animal survival critically depends on the ability to orient in space. Despite their small brains, insects have remarkable capacities for spatial orientation, illustrated by the daily foraging of worker bees [1] or the seasonal long-range migration of monarch butterflies [2]. Goal-directed locomotion in these species serves to purposefully and efficiently bridge distances from meters to thousands of kilometers. Depending on goal and setting, spatial orientation is based on salient landmarks or stable, nearly ubiquitous compass-signals such as the position of the Sun at a given time-of-day [1–5].

**Fig 1:**
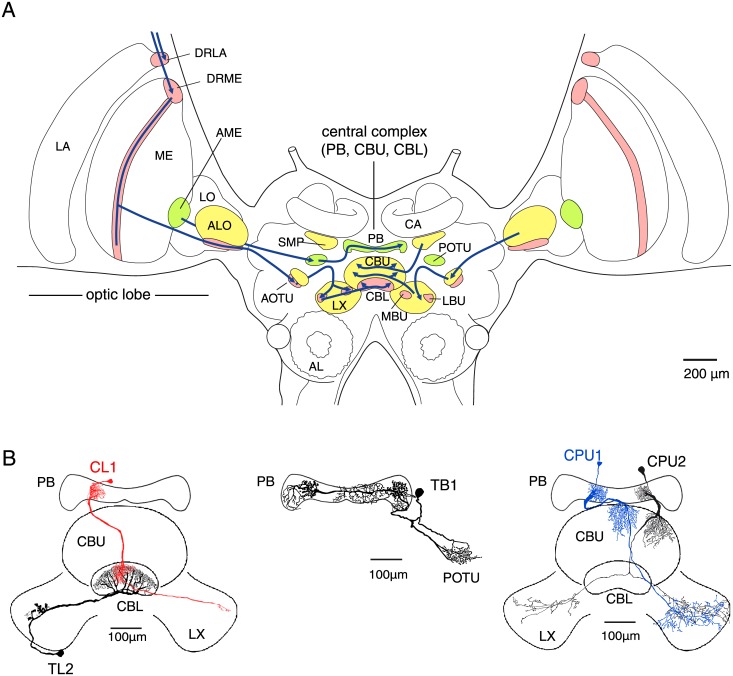
Gross anatomy of the locust brain, visual pathways, and relevant types of central complex neurons. A. Bilateral pathways of light-sensitive neurons from the optic lobes converge onto a network in the central complex (frontal view). Stages of early visual processing include the lamina (LA), medulla (ME) and lobula (LO) of the optic lobe. Neuropils shaded red (green) are involved in an anterior (posterior) pathway of interneurons sensitive to sky compass signals. Additional pathways (yellow neuropils) might signal optic flow and / or represent features of the visual object-background scenery. DRLA (DRME), dorsal rim area of the lamina (medulla); ALO, anterior lobe of the lobula; AME, accessory medulla; AOTU, anterior optic tubercle; POTU, posterior optic tubercle; MBU (LBU) medial (lateral) bulb; LAL, lateral accessory lobe; together with the LAL, the MBU and LBU make up the lateral complex (LX). CBL (CBU) lower (upper) division of the central body; PB, protocerebral bridge; SMP, superior medial protocerebrum; CA, calyx of mushroom body. B. Relevant cell types of the central complex (frontal view). Tangential neurons invade all slices of the CBL (TL neurons) or some slices in the PB (TB1 neurons), as well as regions in a lateral complex (TL) or layers in a POTU (TB). Columnar neurons connect distinct slices of the PB to the CBU (CPU neurons) or CBL (CL neurons) of the central body and have additional arborizations in the lateral complexes. Scale bar, 100 *μ*m.

The central complex in the insect brain is a candidate neural substrate for both strategies of orientation (Fig 1). It plays a major role in higher locomotor control (fly, cockroach) [6,7], visual pattern- and working memory (fly) [8–10], place learning promoted by visual landmarks (fly) [11], and global orientation based on sky compass coding (locust, cricket, monarch butterfly) [12–17]. Its main neuropils are the lower and upper divisions of the central body (CBL and CBU, respectively) and the protocerebral bridge (PB) [18]. These are structured into horizontal layers (CBL, CBU) and vertical slices (PB, CBL, CBU). Two recent studies in the fly *Drosophila* have identified candidate substrates for landmark orientation and idiothetic signaling of head direction in the central complex [19,20]. Neurons that functionally resemble simple cells in mammalian primary visual cortex were found in the fly's homolog of the CBL, whose structural integrity is a prerequisite for the fly's ability for place learning based on visual landmarks [11,19]. Their population response may represent the contour orientation, retinotopic position and movement of bar-shaped objects which flies innately prefer for landmark orientation [19]. Most likely, these representations of putative landmarks are read by cells that connect to the protocerebral bridge [20]. The latter are comparable to vertebrate head-direction cells. Their population response signals the fly's allocentric orientation by exploiting at least two types of cue: the fly's azimuth relative to prominent visual landmarks and, in the absence of the latter, self-motion cues for angular path integration. Hence, cells in the fly's central complex can use idiothetic indications of ego-motion and representations of visual landmarks in a complementary fashion to provide stable signaling of the animal's orientation relative to its local environment.

In the migratory desert locust, allocentric orientation in a more global frame of reference is most likely mediated by "sky compass cells" that indicate the animal's azimuth relative to the Sun. Bilateral pathways from specific areas of the locust visual system converge onto a network in the central complex (Fig 1). Neurons along these pathways and in the central complex are tuned to the polarization pattern of the blue sky and signal the relative position of the Sun even if the latter is not directly visible [12,21]. Neurons that invade distinct slices of the PB show systematic variation in polarization-tuning along the width of the neuropil, resulting in a compass-like representation of heading directions under the open sky mapped onto the slices of the PB [13]. Behavioral data [22] and dynamics of neuronal responses [14] suggest that this compass is read to control heading direction when desert locusts migrate.

By means of intracellular recordings from immobilized locusts, we investigated whether compass neurons at different stages of the central complex network (Fig 1B) also represent the position or any salient feature of possible landmarks, in analogy to observations in the fly "head-direction cells". The respective cells were identified morphologically via fluorescent labelling.

## Materials and Methods

### Experimental animals and preparation

Male adult locusts were obtained from crowded indoor colonies (28°C, 11 h: 13 h light-dark regime). To ease handling during preparation, animals were immobilized via cooling (4°C, 15 min), legs and wings were cut off and the animals waxed to a metal holder. For access to the frontal brain surface, the frons, including antennae and ocelli, was excised, and parts of the subcuticular fat body and tracheal air sacs were removed. Several measures were taken to promote stable recording conditions by reducing movements of the brain. Muscles connected to the antennae and mouthparts as well as the esophagus were transected, the gut was removed through an abdominal incision, and a spoon-shaped wire was waxed ventrally to the head capsule with its loop positioned to support the brain from posterior. Finally, the neural sheath was opened to facilitate brain tissue penetration by the intracellular electrode. Locust saline [23] was applied to replace fatty hemolymph and keep the brain immersed during preparation and recording.

### Intracellular recording

For intracellular recording, two Ag-AgCl wire interfaces were used, one of which served as reference immersed in saline while the other was inserted into a sharp micropipette. Micropipettes were drawn from borosilicate capillaries (0.75 mm ID, 1.5 mm OD, Hilgenberg, Malsfeld, Germany) with a Flaming/Brown filament puller (P-97 Sutter Instrument Company, Novato, CA), and filled with 1 M KCl for electric conduction. Impedances in tissue ranged from 50–200 MΩ. To allow labeling of cells, the tips of the micropipettes were loaded with Neurobiotin tracer (Vector Laboratories, Burlingame, UK, 4% in 1 M KCl) that could be injected iontophoretically (0.5–2 nA, 1–15 min) after recording. Tapped potentials were amplified and band-passed (10×, 20 Hz–20 kHz; SEC 1L/H amplifier, npi electronic, Tamm, Germany) prior to digitization (16 bit / 11.1 kHz; Power1401mkII converter run with Spike2 software, both Cambridge Electronic Devices, Cambridge, UK) and storage. Software for offline analysis was written in MATLAB (MathWorks, Natick, MA, USA).

### Histology

For histological processing, brain preparations were first fixed for 12–24 hrs at 4°C in a solution of 4% paraformaldehyde, 0.25% glutaraldehyde, and 0.25% picric acid in 0.1M phosphate-buffered saline (PBS) and rinsed in PBS (4 x 15 min). Subsequently, intracellular Neurobiotin was coupled to Cy3 fluorophore by means of incubation in a solution of Cy3-conjugated streptavidin (Dianova, Hamburg, Germany, 1:1000) in 0.1M PBS with 0.3% Triton X-100 detergent (PBT) for 3 days at 4°C in the dark. Brains were then rinsed again (PBT, 2 x 30 min and PBS, 3 x 30 min), dehydrated in an ascending ethanol series (H_2_O, 30%, 50%, 70%, 90%, 95%, and 100% ethanol, 15 min each) and cleared in a solution of methyl salicylate in ethanol (1:1, 30–45 min) followed by pure methyl salicylate (45–60 min). Finally, preparations were embedded in Permount (Fisher Scientific, Pittsburgh, PA) and scanned confocally (Leica TCS SP5 confocal laser scanning microscope, Leica Microsystems, Wetzlar, Germany) at 1024 x 1024 pixel resolution and either 10x or 20x magnification (Leica oil immersion objectives HC PL APO 10x/0.40 and HCX PL APO 20x/0.70, respectively). Cy3-fluorescence was induced by excitation at 561 nm (DPSS laser). In most cases relevant neuropils could be identified based on their autofluorescence. AMIRA 5.3.3 (FEI Visualization Sciences Group, Merignac, France) and COREL Photo-paint (X3 V 13.0.0576, Corel Corporation, Ottawa, ON, Canada) were used to generate and edit projection views from confocal image stacks.

### Visual stimulation

**Fig 2:**
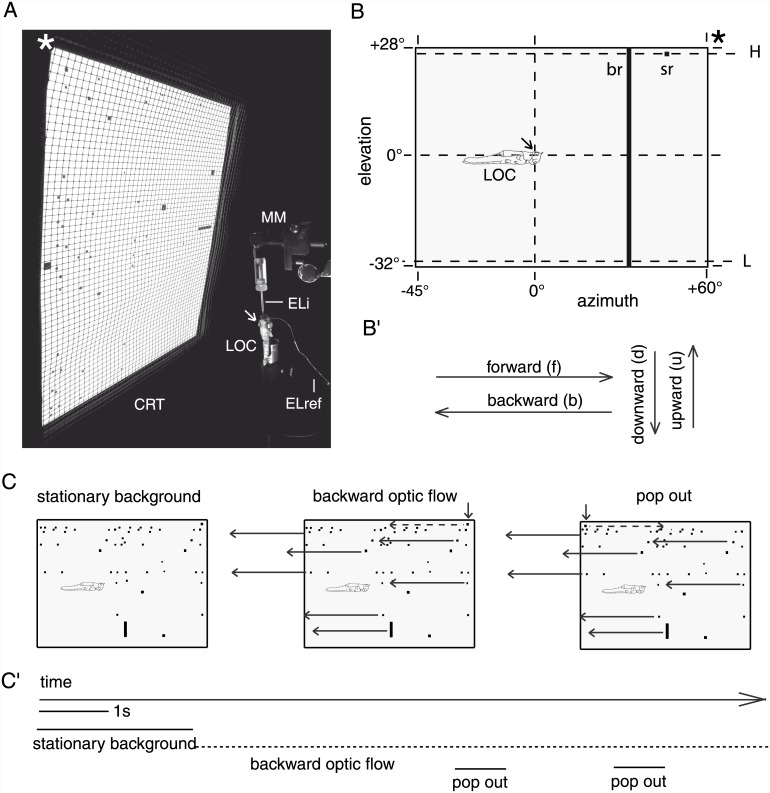
Experimental setup and visual stimulation. A. Experimental setup with display showing stationary background clutter. CRT, cathode ray tube display; asterisk, fronto-dorsal corner mark of CRT; MM, micromanipulator for positioning of intracellular electrode; ELi, intracellular electrode; ELref, reference electrode; LOC, locust (viewed from dorsal); arrow, stimulated (left) eye. Note the slight tilt of the CRT and the mesh wire shielding which prevented inductive interference between display and measurement circuit. B. Region in the left antero-lateral visual field covered by the stimulus display. Arrow, position of stimulated (left) eye; asterisk, fronto-dorsal corner mark of CRT; LOC, locust (viewed from lateral). Dashed horizontal lines tagged H and L indicate high- and low-elevation trajectories for translations of single small-field squares. sr, br: commonly applied square- and bar-shaped stimulus; about 2°x1.5° and 2°x60°, respectively. Albeit both types of stimulus are shown together here, they were never presented simultaneously. B'. Terminology for directions of translational motion, illustrated according to the orientation of the diagram in B. Abbreviations are given in parentheses. Forward (backward) direction of translation is abbreviated "f" ("b") and high (low) elevation is abbreviated "H" ("L"). Thus, the term "b, L" ("f, H") refers to the backward (forward) translation of a 2°x1.5° square -29.5° (+25.5°) in elevation. See S1 Fig for commonly applied sequences of such simple stimuli. C, C'. Components (C) and time course (C') of a more complex stimulus sequence designed to mimic sudden forward small-field motion against a backward optic-background flow. The sequence begins with a stationary display of the "background clutter" pattern, followed by repeated backward translations of the entire pattern (indicated by horizontal arrows) in an optic-flow like manner at 70°/s (dashed horizontal line in C'). In the late phase of the sequence, an individual object in the background flow (short vertical arrows in C) pops out of it twice by changing its direction of motion, i.e. the object suddenly moved forward against the backward background flow (absolute velocity 80°/s; velocity relative to background flow -150°/s). The object, a black, rectangle square of about 2° visual angle azimuthal extent and 1.5° elevational extent, is identical to the one used in the simple stimulus regimes and moves at the same high elevation of 25.5° also applied in the latter. For further details on stimuli, see Materials and Methods.

We screened sky compass neurons in the central complex of the locust brain for responsiveness to various visual entities, including object size, vertical compactness, contour orientation, egocentric object position, and rotational / translational motion. Most common stimuli included small-field and wide-field elements as well as stationary display and simulated translational, rotational and progressive motion (Fig 2 and S1 Fig).

Neural activity was recorded in a Faraday cage open to one side. All light sources outside the cage were covered with red filters to prevent interference with controlled stimulation. Visual stimuli were generated using a ViSaGe device (Cambridge Research Systems, Rochester, Kent, UK) and displayed on a 22” CRT screen (DP2070SB, Mitsubishi, Tokyo, Japan) positioned slightly tilted as to cover -45° to 60° in azimuth and -32° to 28° in elevation within the left antero-lateral visual field (Fig 2A and 2B). This visual subfield covered by the CRT is hereafter referred to as the mapping field. With the brightest illumination applied here (RGB 255-255-255), the average luminance of the light emitted from the entire screen was 89.4 cd/m^2^ (900 measurements evenly spaced across the display, using the OPTICAL photometer provided by Cambridge Research Systems; standard deviation 5.14 cd/m^2^). A "neutral, grey background" used for measurement of background activity as well as to display the main types of bar- and square stimulus against was generated by setting the screen to RGB 127-127-127. All visual stimuli were generated by custom-written MATLAB functions using the Cambridge Research Systems toolbox. These also delivered trigger signals for offline analysis of raw data in MATLAB and provided documented pseudo-randomization of order within most stimulus sequences.

Initially, we screened central-complex neurons for responsiveness to basic visual entities, such as object features or egocentric object location. To this end, we designed a variety of visual stimuli, some of which were skipped after a few recordings. Flashes of blue, red, green, and white light comparable in photon flux rate (photon flux measured at 710 nm, 520 nm and 450 nm varied from 7·10^13^ to 9·10^13^ photons·s^-1^cm^-2^ at each of the three wavelengths) served to test for general sensitivity to light and for spectral tuning. Photon flux was measured with a digital spectrometer (USB2000, Ocean Optics Inc., FL, USA) with the detector head at the position of the compound eye, directed toward the CRT display. Black (white) squares were displayed against white (black) background at pseudo-randomized positions across the entire mapping field to characterize any spatial tuning within it. Groups of "random squares" approaching (looming) the locust from the center of the frontal visual field (generated using the built-in function of the Cambridge Research Systems toolbox), as well as translating sine gratings with different spatial frequencies, angular extents, and angular orientations were displayed to simulate both translational and rotational wide-field motion. Stationary displays of these and any other moving stimuli served to control for mere responsiveness to changes in ambient light level or spatial tuning within the mapping field in case a receptive field mapping had not been performed. These stimuli did not drive any of the cells encountered in a manner tuned to object position or wide-field motion. At most, they evoked transient responses to rapid changes in ambient light level that are, however, not further characterized in the results section. Additional stimuli designed to mimic black or white bar-shaped objects were displayed individually and varied in vertical extent, contour orientation and combinations thereof. These were presented translating with different velocities along different horizontal (forward or backward motion) or vertical (upward or downward motion) trajectories with stationary controls applied in some experiments. In particular, black, rectangular small-field squares (about 2° visual angle horizontal extent and 1.5° vertical extent) proved an adequate stimulus to the cells considered here (Fig 2B). These small-field squares hence became the building blocks of additional stimulus sequences for deeper characterization of the responses that followed the pre-screening (S1 Fig).

### Features and abbreviations of basic stimuli

Most commonly, forward and / or backward translations of a black, rectangular small-field square against a grey background at either high elevation (+25.5°) or low elevation (-29.5°) were combined in different sequences (S1 Fig). The angular velocity against the blank background was 70°/s (on average, see below), and trajectories spanned the entire width of the display (-45° to + 60° in azimuth). Forward (backward) directions of translation are abbreviated "f" ("b") and high (low) elevations are abbreviated "H" ("L"). Therefore, the term "b, L" refers to the backward translation of a 2°x1.5° square at 70°/s, from +60° to -45° in azimuth at -29.5° in elevation. More precisely, the velocity of the moving square *on the CRT* display was constant. The horizontal translation at 70°/s corresponded to about 1.5 s stimulus duration. In short sequences of two stimuli that were used in the early experimental phase (S1A and S1B Fig), the inter-stimulus interval was about 1.3 seconds, while it was about 500 ms for the more commonly applied longer sequences (S1C and S1C' Fig). As a consequence of the display's flat geometry, the angular velocity seen by the animal slightly increased (decreased) as the square moved toward (away) from the animal. As the same holds for angular size, the translating squares also had a mild looming component. However, the response behavior we observed strongly speaks against a role of these minor changes of angular velocity and size. In addition to this simple stimulus regime, a more complex stimulus-background scenario was applied in some cases (Fig 2C and 2C').

### Basic offline data analysis

Spikes were detected by threshold based event detection. An upper threshold as well as an absolute refractory period of 1 ms were applied to prevent false positives. For non-smoothing visualization of spiking dynamics, we applied the instantaneous interspike interval (ISI) method [24] instead of the classical peristimulus time histogram (PSTH) to avoid both arbitrariness of bin placement and the problem of bin width optimization.

### Criteria for inclusion in final analysis

Physiological data were only included in the final analysis if the recorded neuron was identified. Ideally, this was provided by distinct labeling of an individual cell. If more than one neuronal cell were labeled in the same preparation, recordings were assigned to morphologies based on characteristic patterns of background activity that had previously been determined for distinct cell types (for details, see [14]).

### Rating responsiveness and response amplitude

Most types of central-complex neuron encountered here exhibited a background activity that was relatively high in average rate and marked by cell-type specific dynamics [14]. Concurrently, putative responses were occasionally subtle, standing out against the complex patterns of background activity rather in terms of their consistency across trials than by high steps in firing rate. Hence, for decision on inclusion of putative responses in meta-analyses, we prioritized consistency across several trials recorded from the same cell or between several cells of the same type. For further analysis, response spike rates were calculated based on spike counts in the first 1350 ms after the stimulus began. Prior to pooling response data obtained from several cells, we normalized these rates for each trial by subtracting the spike rate observed in a window of 450 ms directly preceding the stimulus. For comparing these normalized response spike-rates between conditions, we used non-parametric hypothesis testing (Wilcoxon rank-sum test, two-sided sign test) as box plots indicated non-normal distributions and / or unequal variances. To investigate more carefully whether short- and long-term dynamics of background activity might have modulated responses, we investigated how responses to the first stimulus (I) relate to the local state of background activity that directly precedes the stimulus presentation and (II) compare to different levels of background activity sampled over prolonged periods (commonly several minutes, scattered throughout the course of the experiment, bin size for calculation of spike count distributions 1000 ms). For (I), we performed a linear regression on the spike counts observed during different peristimulus time windows: the last 700 ms prior to stimulation as opposed to either the first or second 700 ms window during presentation of the standard translating-square stimulus which had a duration of about 1400 ms in total. This procedure is reminiscent of the first steps in the analysis of evoked activity in V1 neurons reported by Arieli et al. [25]. It was confined to responses pooled across CPU1 and CPU2 neurons, as these comprised a sufficient number of cells and repetitions, and both types of neuron showed pronounced local dynamics of background activity, thus producing a sufficient span of spike counts for a meaningful analysis of covariance. For (II), spike counts in stimulus time-windows were compared to different relevant quantiles of the background activity spike count distribution (based on 1s bin width), similar to the procedure described in [14].

### Data plots

Raster plots were generated using the MATLAB code kindly provided by Rajiv Narayan, Boston University (rasterplot.m, provided at www.mathworks.com). Plots of spike count distributions and ISI histograms were based on code kindly provided by Prof. Dr. Sonja Grün (Research Center Jülich, RWTH Aachen University). In box plots, boxes span the inner 50% of the respective distribution from the first to the third quartile. Box notches give the 95% confidence interval of the median, i.e. two median values differ significantly (at α = 0.05) if the notches of their boxes do not overlap. Whiskers extend to the adjacent values, which are the most extreme data values that are not considered outliers. Outliers are shown in cross-shaped markers. More detailed descriptions of distributions are provided by scatter plots that use marker size to indicate frequency of observation (bubble plots). In these, the diameters of the circular markers are linearly scaled to the absolute frequency at which a value defined by the center of the marker was observed. In contrast to box plots, scatter plots can visualize features such as the actual shape of symmetrical distributions (e.g., to distinguish between unimodal and bimodal cases) and do not involve statistics that should be avoided for low sampling sizes, such as median values or quartile ranges. For visualization of the time course of responses, we used plots of the *normalized* instantaneous ISI. To this end, the ISIs of each individual response were first transformed to the interval [0 1] by element-wise subtraction of the minimum ISI and subsequent division by the maximum of the resultant values. In case of repeated presentation of the same sequence, the median normalized ISIs of the individual responses was calculated. As a consequence, average values near zero or unity indicate high consistency of response courses over trials. For the sake of intuitive illustration, we added plots of trial-averaged Gaussian-smoothed PSTHs to the raster plots of some exemplary responses (based on Jude Mitchell's MATLAB function compute_gauss_smooth, accessible at http://www.snl.salk.edu/~jude/sfn2008/index.html; adopted by Kreuz et al. [24].

## Results

### Dataset

Fig 1B illustrates the morphological cell types encountered in the present study. Columnar neurons connect distinct slices of the PB to the CBU (CPU1- and CPU2 neurons) or CBL (CL1 neurons) and have additional branches in the lateral complexes, the main input- and output relays of the central complex [18]. Tangential neurons invade all slices of the CBL (TL neurons) or many slices in the PB (TB1 neurons). The putative processing hierarchy is TL-CL-TB-CPU [26]. Data included in the final analysis covered 17 neurons from 17 adult gregarious animals. Of these, 3 recordings were from CL1 neurons, 4 from TB1 neurons, 5 from CPU1 neurons and 5 from CPU2 neurons. Datasets may vary between figures due to the purpose and demands of the respective analyses. Measurements of polarization sensitivity were performed in the same cells to confirm their role as compass cells [14].

### Polarization-sensitive neurons of the central complex signal small-field motion in a novelty-dependent manner

**Fig 3:**
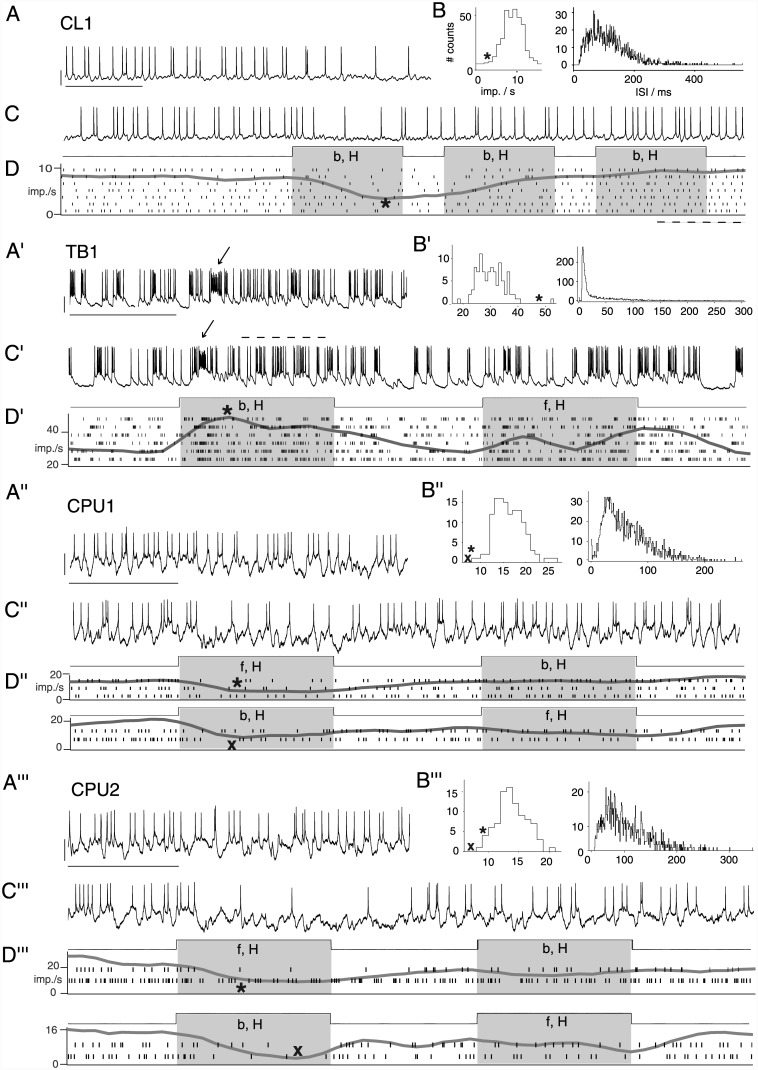
Background activity and responses to small-field motion in four types of central-complex neuron. Subfigures show data from a CL1 neuron (A-D), TB1 neuron (A'-D'), CPU1 neuron (A''-D'') and CPU2 neuron (A'''-D''') of the central complex. A-A'''. Traces illustrating background activity. B-B'''. Spike count distribution and ISI histogram of background activity (trial duration for spike count 1 s). Asterisks and x-marks indicate the location of spike rates observed at the peak (or nadir) of the trial-averaged responses shown in D-D'''. C-C'''. Responses to presentations of a translating black, rectangular small-field square (about 2° visual angle azimuthal extent and 1.5° elevational extent) against a blank background showed fast adaptation to motion along the same trajectory. Stimulus types and periods, indicated below the recording traces, also hold for raster plots in D-D''', unless indicated otherwise. Stimulus abbreviations: f (b), forward (backward) small-field translation; H (L) trajectory with high (low) elevation. D-D'''. Raster plots and trial-averaged, Gaussian-smoothed PSTHs of additional responses. Spike rates occurring at the peak (or nadir) of the averaged response (marked by asterisks or x-marks) are located at the edges of the spike count distribution obtained from long-term background activity (see B-B'''). This indicates that responsiveness is robust across the trials shown here, but response amplitudes vary and individual responses resemble sections of background activity. In particular, arrows in A' and C' mark prominent bursts in background activity and in the phasic response period for the TB-neuron. In the two subtypes of CPU neuron (D'' and D'''), responses are independent from direction of motion. Dashed lines indicate periods of rebound-like increase in spike rate in D and a period of tonic increase in spike rate in C'. Bars, 1 s; 10 mV.

As previously reported [14], all cells showed background activity with pronounced cell-type specific dynamics (Fig 3A–3A''' and 3B–3B'''). When tested for responsiveness to virtual objects, neither topographic mapping of object positions across the population of cells, nor narrow tuning to object features was observed. CL1, TB1, CPU1 and CPU2 neurons responded to moving small-field squares (Fig 2B and 2B', S1 Fig) or compact bars but not to visual flow (Fig 2C, middle). TL2 cells [27] showed no responses at all (n = 4; N = 15, 27, 10 and 50 presentations of a moving small-field square; N = 6, 3 presentations of moving bars in two of the cells; data not shown). Fig 3 shows selected responses of the other cell types to translational motion of a black small-field square (about 2°×1.5° in visual angle) against a uniform grey background (Fig 3C–3C''' and 3D–3D'''). In addition, some CL1 neurons showed similar responses (data not shown) to presentations of a single, translating bar spanning the height of the stimulus display (Fig 2B). The sign of the responses was consistent across cells of the same type: TB1 cells increased firing, while CL1 and CPU cells responded with inhibition. The time course of responses also appeared to depend on cell type. In CL1 neurons, responses often outlasted the duration (about 1.5 s) of the first stimulus in a sequence and tended to be followed by rebound-like states of mildly (Fig 3D) to substantially increased spiking. TB1 neurons mostly responded less prominently by bursting to the onset of motion. An example of more prominent responses that included a moderate, tonic increase in spike rate is shown in Fig 3C' and 3D'. Similarly, both phasic and prolonged responses were found in CPU1- (Fig 3A''–3D'') and CPU2 (Fig 3A'''–3D''') neurons. In each cell-type, responses—even those obtained from the same cell—furthermore varied in latency and amplitude, as evident from the raster plots in Fig 3. In addition to the variability of response amplitudes, we observed occasional response-like spiking in the background activity of all cell types (Fig 3A–3A'''). Nevertheless, spike rates at the peak (or nadir) of the trial-averaged responses (Fig 3D–3D''') differed substantially from the dominant level of background activity in the respective cell (Fig 3B–3B'''). A more pronounced variability in response amplitude was observed in CPU cells (Fig 3D'' and 3D'''), consistent with previous observations on their responses to polarized light [14].

**Fig 4:**
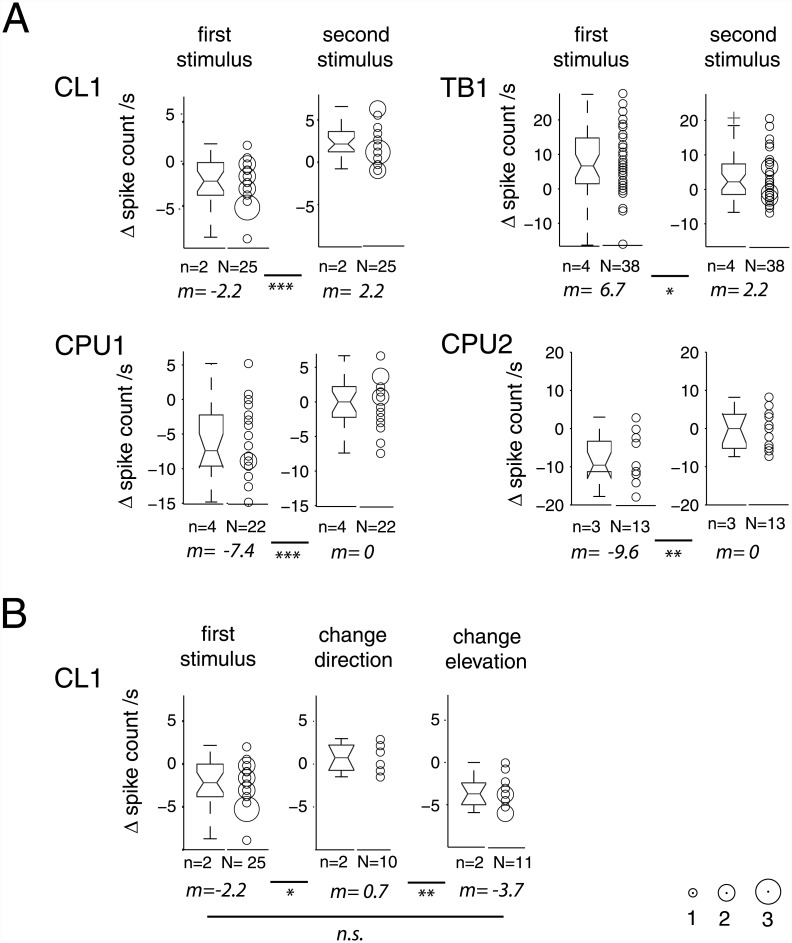
Stimulus-specific adaptation of responses to small-field motion in CL-, TB- and CPU neurons. A. Responses to small-field motion are marked by rapid adaptation between the first and second presentation of a single moving small-field square. Box- and bubble plots show differences in spike count between stimulus time-windows and background activity in a period of 450 ms preceding stimulation, based on N responses obtained from n neurons. Notches in box plots indicate 95% confidence interval of the median and circular plot markers in bubble plots are scaled to the frequency of observations (see scale, bottom right). Some outliers were truncated for the sake of better visualization. *m*, median; asterisks: level of statistical significance (Bonferroni-corrected) of difference in median as calculated by Wilcoxon rank-sum tests (* *p*_*corr*_<0.05, ** *p*_*corr*_<0.01, *** *p*_*corr*_<0.001). TB1 cells showed excitatory responses to the first stimulus in a sequence, while all other types of neuron showed inhibition. Note the increased spike rate of CL1 cells during presentation of the second stimulus, which is related to rebound-spiking after the inhibitory response to the first stimulus (see Fig 3D). Data include many cases where first and second stimulus differed in direction of motion (see Table 1). Nevertheless, pronounced adaptation occurs between the first and second stimulus. B. In many cases, adaptation could be reversed by switching the elevation of a horizontal trajectory, as illustrated here for population data of CL1 neurons sufficient in sample size for statistical testing.

In repeated stimulations, responses to the first stimulus were independent of the particular position of the stimulus trajectory as well as of the direction of motion (Fig 3D'' and 3D'''; see also S1 Fig) along that trajectory. Fig 4A shows pooled data on the effects of repeated stimulation. In all cell types, the successive presentation of two stimuli resulted in rapid adaptation, as illustrated by the responses in Fig 3 before. This links responsiveness to stimulus history: responses were strongest for the first presentation of a small-field motion stimulus that had been absent for an extended period, and occasionally responsiveness was totally confined to these "novelty" trials (Fig 4A). The exact effect of pause duration was not explored.

**Fig 5:**
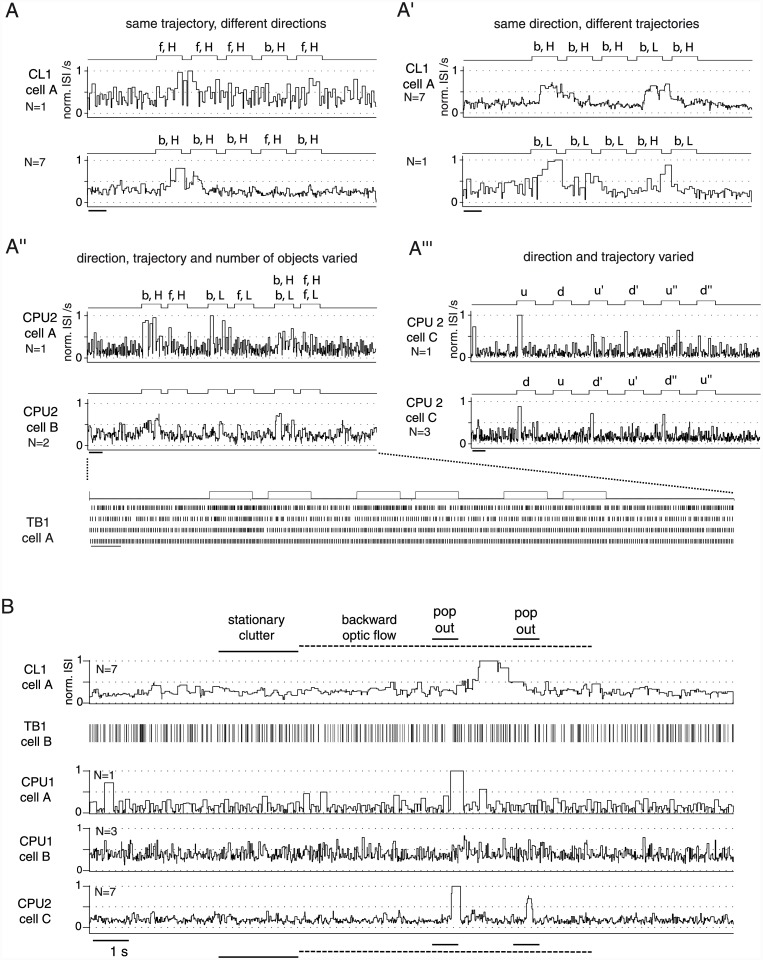
Context dependent responses to moving objects. The courses of N responses to a respective stimulus sequence are visualized for selected neurons by normalized instantaneous interspike intervals (norm. ISI), averaged across trials when N > 1. For a TB1 neuron, raster plots were preferred. Values of 0 or 1 indicate highest consistency in response course across trials. A-A'''. Each subplot represents N responses to a specific stimulus sequence, obtained from cells identified by capital letters within each cell type (compare to subfigure B). A-A'': Horizontal trajectories; forward (f) or backward (b) motion at high (H) or low (L) elevation. In A'', the third and fourth stimulus consist of two objects that move in the same direction but at different elevations. A''', Responses to upward (u) and downward (d) motion along vertical trajectories at different azimuths (-38°, 7.5° and 54°). Here, the transitions from d to u' and d' to u'' correspond to switches in the azimuth of the trajectory, whereas the particular sequence of azimuths was randomized in each trial. As a consequence, the lower subplot shows data pooled across 3 different chronological orders of azimuths. For both vertical and horizontal trajectories, it is the switch in trajectory that triggers responses, not the particular position of the trajectory or changes of the direction of motion. Bars, 1 s. B. Responses of the same types of neuron are also suited to detect a distinct object that changes its direction of motion (pop out) against a moving background of identical and very similar objects. For detailed description of the stimulus sequence, see Fig 2.

The rapid adaptation is evident in the pooled data shown in Fig 4A despite the fact that the datasets include many cases in which the first and second stimulus differed in direction of motion (Table 1). This suggests that the adaptation was unaffected by changes in the *direction* of motion within the *same* region of the visual subfield. A differentiated, qualitative inspection of the raw data confirms this interpretation (S2 Fig). In addition to stimulus sequences that included changes in direction of motion (from forward to backward or vice versa; in few tests from downward to upward or vice versa), we applied switches of the trajectory's position in the visual subfield by varying its elevation or, in few tests with upward / downward motion, its azimuth. Such changes of the stimulus region reversed the adaptation in many cases, as evident from the population data for CL1 neurons shown in Fig 4B and Table 2 as well as from the responses of CL1, TB1 and CPU2 cells shown in Fig 5A–5A'''. Fig 5 includes plots of the instantaneous, normalized interspike intervals (ISI). This kind of plot was particularly suited for the inhibitory responses of CL1 and CPU cells and, as raster plots are, is unaffected by the problem of bin width and bin positioning that complicates conventional peristimulus rate histograms. For data pooled across repeated stimulations (N>1 trials), normalized ISI values of 1 (or 0) indicate an inhibitory (or excitatory) response of absolute consistency in nadir (peak) position across trials. The substantially stronger effect of changing trajectories as opposed to changing direction of motion (Fig 5A–5A''') suggests a stimulus specific adaptation with respect to the region of the visual field that is spanned by the stimulus trajectory. This response feature could serve to signal the novel appearance of a moving small-field object, but not a mere change in the behavior of an object, i.e. here, a change in the direction of motion.

**Table 1:** Statistics of data distributions shown in Fig 4A.

cell type	n	N_total_	N_dif dir_	W-*p*	W-*p*_corr_	W-Z	W-sum
**CL1**	2	25	3	1.2*10^-7^	3.6*10^-7^	-5.3	364
**TB1**	4	38	32	0.0144		2.45	1699
**CPU1**	4	22	18	9.7*10^-5^		-3.9	328.5
**CPU2**	3	13	8	0.0048		-2.82	120

n, number of cells; N_total_, total number of evaluated responses to first and second stimulus; N_dif dir_, number of cases in which the first and second stimulus differed in direction of motion; W-*p*, W-*p*_corr_, W-Z and W-sum, *p*-value, Bonferroni-corrected *p*-value, Z-statistic and rank-sum, Wilcoxon rank-sum test. Note that correction for multiple testing includes comparisons shown in Fig 4B, but not those in Fig 6C as the latter were obtained by comparison to global dynamics of background activity, not to local precedent background activity.

**Table 2:** Statistics of data distributions shown in Fig 4B.

comparison	n	N	W-*p*	W-*p*_corr_	W-Z	W-sum
**1** ^**st**^ **stimulus vs. change direction**	2, 2	25, 10	0.0058	0.0174	-2.76	374
**change direction vs. change elevation**	2, 2	10, 11	0.0011	0.0022	3.28	157
**1** ^**st**^ **stimulus vs. change elevation**	2, 2	25, 11	0.18		1.56	508.5

n, number of cells; N, respective number of responses evaluated; W-*p*, W-*p*_corr_, W-Z and W-sum, *p*-value, Bonferroni-corrected *p*-value, Z-statistic and rank-sum, Wilcoxon rank-sum test. Note that correction for multiple testing also includes comparisons shown in Fig 4A.

All responses hitherto mentioned were obtained with small-field objects moving against a blank, uniformly grey background. We wondered whether the novelty-detection capacity of these cells extends to more complex object-background scenarios as well. To test this, we introduced a more complex stimulus that included a group of squares and a bar translating backwards along parallel trajectories at a common velocity (70°/s) to simulate a unilateral visual flow as resultant from a rotational ego-motion of the locust (Fig 2C and 2C'). After an initial stationary presentation of the background clutter and several repeated translations, the behavior of an individual square was suddenly altered as to render it a distinct object that suddenly pops out against the uniform visual flow. More precisely, we applied a change in the direction of motion of the individual square which results in motion relative to the background clutter. All elements of the background clutter had the same contrast as the distinct square. The sudden transition to an opponent motion against the background clutter (the "pop out" event) triggered responses in this object-background regime (Fig 5B). It is crucial to recall here that changes in direction of motion did *not* break the stimulus specific adaptation in the simple "single square against blank background" regime (Fig 5A–5A'''). One might argue that motion of the individual square in a direction opposite to the optic flow results in an increased relative velocity of the target-like object which, in principle, could have promoted target detection as well. However, a mere increase in relative velocity by faster translation (140°/s) in the same direction as the optic-background flow (70°/s), that was applied as a control condition in the same CL1 neuron included in Fig 5B, did not trigger any responses (5 trials, data not shown). As previously observed for single-square responses, CL1 cells as well as the CPU1/2 neurons responded to the pop out with inhibition (reflected by an *increase* in interspike interval), whereas the individual putative response of the TB1 cell was excitatory. Again, some sections of background activity resemble the responses, which is particularly evident in the raster plot of the TB1 response. Yet, repeated stimulations (CL1 cell A, CPU2 cell C; both N = 7) evoked consistent responses to the pop out whereas peristimulus background activity showed little consistency across trials. This is again reflected by the average normalized interspike intervals, which reached values of 1 in response to the pop-out, whereas values of peristimulus background activity roughly spanned 0.25–0.5 (Fig 5B). The different response amplitudes of CPU1 cell A and B reflect the relatively high level of response-variability that was previously observed for responses to simple stimuli.

### Response amplitudes are modulated by states of dynamic background activity

The amplitudes of initial responses co-varied with preceding states of background activity (Fig 6A). High-level background activity might, therefore, mask inhibitory responses (CL1, CPU1) by superimposition and reduce the relative strength of excitatory responses (TB1) as has been observed in cat visual cortex [25]. To quantify the association between responses and precedent background activity, we tested for correlation between binned spike counts (700 ms windows) during stimulus presentation (first-stimulus responses only) and during the directly preceding stimulus-free period in CPU neurons. The analysis revealed a highly significant and strong correlation with about 70% shared variance (Fig 6B). Yet, the regression line for spike counts obtained from the second half of the stimulus period is slightly closer to the bisecting line of the plot, reflecting the transient course of these responses. The plots illustrate substantial variability in response amplitude, both for the early and the late phase of responses and between recordings as well as across the course of a respective recording. In particular, data points near the bisecting line that represent trials with no pronounced response were often obtained from recordings that included strong responses as well. The results confirm that responses of CPU neurons were co-shaped by the state of immediately preceding background activity.

**Fig 6:**
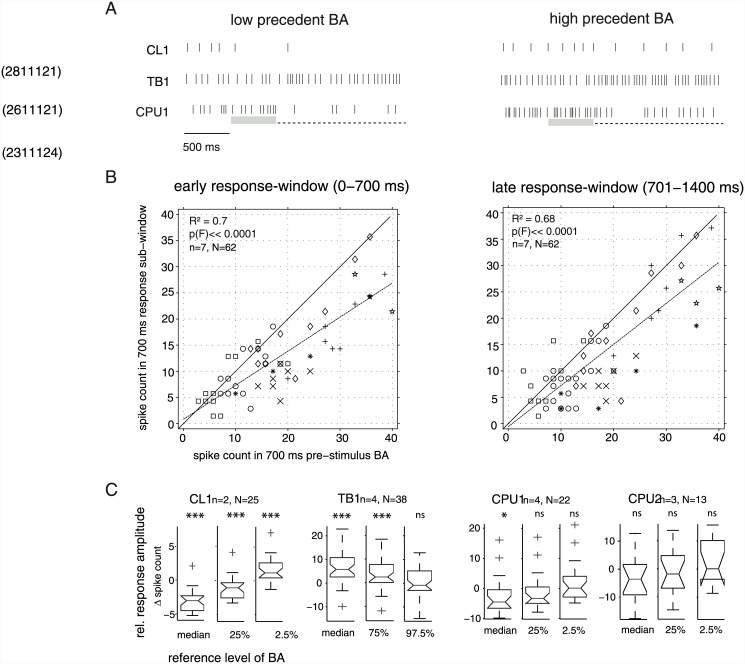
Modulation of responses to moving small-field objects by variable background activity. A. Raster plots show selected responses to a translating small-field square (dashed line: stimulus window) which are modulated by relatively low states (left subplot) and relatively high states (right subplot) of precedent background activity (BA). The relevant period of background activity may roughly cover the last 500 ms (grey shading) preceding the stimulus. B. Correlation analysis in CPU neurons. Spike counts within the first (second) 700 ms-window during stimulus presentation were plotted against those in the 700 ms-window of background activity directly preceding the stimulus. Data points close to or above the bisecting line correspond to trials that lacked the inhibitory response typical for CPU neurons; strongest responses are reflected by data points closest to the x-axis. Data were obtained from 3 CPU1- and 4 CPU2 neurons and cover 62 responses to the first stimulus in sequences of single small-field squares translating against a blank background; different plot marks correspond to different neurons. Plot marks are not scaled to the frequency of observations, thus identical values from the same experiment appear as a single data point. Dotted lines show linear regressions. C. Response amplitudes compared to different states of background activity. Box plots show distributions of relative response amplitudes (changes in spike rate relative to different levels of background activity). These were calculated for responses to the first stimulus in a respective sequence of small-field squares translating against a blank background (N responses from n neurons). For estimation of relative response amplitudes, we subtracted the spike rates of different levels of background activity (1s bins), obtained from the same respective cell throughout the entire experiment. These levels include the median and two additional quantiles of the background activity's spike count distribution that lie beyond the median in the same side of the distribution as expected for responses. In case of the inhibitory responses in CL- and CPU neurons, the 25% and 2.5% quantile of the spike rate distribution were used. For the excitatory responses of TB neurons, the 75% and 97.5% quantile provide relevant normalization. Indicated above each box-plot is the result of a two-sided sign test for zero median (* *p*_*corr*_<0.05, ** *p*_*corr*_<0.01, *** *p*_*corr*_<0.001, ns not significant). Note that a non-significant test result here does not imply that responses did not involve a significant change in spike rate as compared to the local background activity that *actually* preceded the responses (see Fig 4A). While responses are relatively robust compared to the median levels of background activity (left columns of subplots), the additional response-type specific normalizations (middle and right columns) reveal that more extreme levels of background activity may result in masking of responses, as the difference to zero change decreases substantially and may become non-significant. Notches in box plots indicate 95% confidence interval of the median. Some outliers were truncated for the sake of better visualization.

We also assessed the degree to which this state-dependency could actually mask responses in each of the cell types encountered here. To this end, we compared spike counts of unadapted responses to different states of long-term background activity, measured at different points in time throughout an experiment (see Materials and Methods). The results imply that background activities in each type of neuron included states suited to completely mask even the most pronounced responses observed here (Fig 6C and Table 3). For instance, the relatively strong responses of CL1 neurons appeared rather neutral when compared against the lower quartile of the background activity's spike count distribution and even corresponded to an *increase* in spike rate relative to the lower extreme (2.5^th^ percentile) of background activity.

**Table 3:** Statistics of data distributions shown in Fig 6c.

cell type	n	N	BA _ref_	*p*	sign
**CL1**	2	25	median	1.5*10^-6^	1
**CL1**	2	25	25%	0.00016	3
**CL1**	2	25	2.5%	0.00091	21
**TB1**	4	38	median	6*10^-7^	34
**TB1**	4	38	75%	0.00047	30
**TB1**	4	38	97.5%	0.6271	17
**CPU1**	4	22	median	0.0169	5
**CPU1**	4	22	25%	0.1338	7
**CPU1**	4	22	2.5%	1	11
**CPU2**	3	13	median	0.2668	4
**CPU2**	3	13	25%	0.5811	5
**CPU2**	3	13	2.5%	1	7

n, number of cells; N, respective number of responses evaluated; BA _ref_, reference level of background activity used for data normalization; *p* and sign, *p*-value and sign-value of two-sided sign test for zero median.

## Discussion

We characterized how central-complex neurons of the locust brain, known for their role as sky compass cells, respond to dark virtual objects moving through a unilateral part of the visual field. In sequential translations of a single small-field square, initial responses were independent of direction of motion and trajectory but showed strong and rapid adaptation. Changes in the direction of motion, suited to mimic a change in behavior of the *same* object (or an apparent change as a result of rotational ego-motion) did not reverse the adaptation. By contrast, switching the trajectory of motion, corresponding to the appearance of a *novel* object concurrent with the disappearance of the previously presented one, dis-adapted the cells in many cases, strongly suggesting region-specific adaptation. This confirms and complements previous observations [28]. In vertebrates, stimulus-specific adaptation is a prominent feature of higher sensory processing, a presumed correlate of behavioral habituation to frequent sensory input and closely related to the capacity of novelty detection in the sensory scene [29,30]. We also show that the direction unselective cells encountered here can detect moving small-field targets against an opposing optic background-flow of objects similar or identical to the "target". This response behavior seems to be distinct from the responses of more peripheral cells in the dragonfly brain [31,32]. Those neurons signal the motion of small targets (1°-2°) based on selectivity for object size, direction of motion and high contrast against the entire visual background.

The responses of cells we encountered in the locust central complex were marked by a prominent context-dependency. In particular, contextuality was mediated by stimulus (region)-specific adaptation and a possible increase in target saliency by the integration of concurrent input from the visual background scenery. In more general terms, contextuality is reflected by the fact that these cells are at least bimodal, signaling novelty events in the visual scenery and sky-compass information [12,28,33].

In addition to stimulus-specific adaptation, responses in both modalities correlate with the state of background activity that can effectively mask them [14]. Similarly, the large variability of visual responses in cat V1 neurons has been explained in terms of an integration of prestimulus background activity with an ideal (trial-averaged) representation of a visual stimulus [25]. This phenomenon has been related to attention and perceptual bias or expectancy, as it correlated with stimulus-detection rate in monkey vision [34] as well as perceptual decisions on ambiguous stimuli in both human vision [35] and human somatosensation [36]. In central-complex neurons in the locust brain, masking by prestimulus activity might also serve the gating of sensory responses depending on the acute relevance of attending to a respective type of information, e.g. object motion or sky-compass signals [14]. In line with this idea, compass responses to polarized light were also occasionally masked by high-level background activity in CPU neurons, i.e. at the output stage of the network [14]. Homberg [37] demonstrated that changes in the background activity of CPU cells may relate to locomotor state based on recordings in tethered flying locusts. Such an effect could modulate the responsiveness to object stimuli used in the present study depending on locomotor state, which could not be addressed here as a result of the invasive preparation. Still, it seems unlikely that this would generally "switch off" the responses observed here and in [28] as they could signal critical events, e.g. the appearance of an in-flight predator or imminent collision in a dense swarm. Escape in response to such events is mediated by fast peripheral pathways that bypass the central complex and include cells which respond to looming and translating objects with region-specific as well as object-size-specific adaptation [38–43]. A relay of such responses to the central complex, i.e. to a central integration-site involved in higher locomotor control, might serve to mediate between the acute necessity to escape from a threat (predation, collision), and the higher goal to locomote along a certain compass course. Such an interaction could be explored using combined stimulation with moving objects and polarized light.

In contrast to our observations in the locust, tangential and columnar cells in the *Drosophila* central complex were recently shown to signal allocentric orientation by representing and tracking visual landmarks and via integration of self-motion cues in darkness [20]. The respective cell types are morphologically similar to TL- and CL neurons, and their response features are suited to mediate spatial orientation in structured local settings, a capacity demonstrated in flies [11,44] and crickets [45]. The difference to our physiological observations in the locust might relate to a difference in lifestyle. *Drosophila* largely lives in local, visually rich environments suited for landmark learning. In contrast, the desert locust is a long-range migrating species that might preferentially rely on compass navigation. In addition, flight in a dense swarm, typical for gregarious locusts, may necessitate sensitivity of the steering-control system to object approach for the purpose of collision avoidance. In contrast, locusts in their solitarious phase, which have a life style more similar to flies, might be expected to more strongly rely on landmark learning and representation.

Although *Drosophila* can also navigate using the sky polarization pattern [46] and occasionally travels long distances [47], neurons that might mediate polarotactic behavior have not been described yet. On the other hand, the allocentric coding of heading directions based on landmarks as shown in *Drosophila* likely requires active locomotion of the animal and self-generated sensory feedback from the visual and antennal system, which were not available in our experiments on restrained locusts. Future experiments both in *Drosophila* and *Schistocerca* are therefore necessary to reveal how landmark and sky compass based orientation coding is possibly integrated and whether substantial species-specific differences indeed exist.

## Supporting Information

S1 Fig Commonly applied sequences of simple stimuli. All stimulus sequences shown here are based on a translating small-field patch and a uniform grey background (70°/s; corresponding to about 1.5 s stimulus duration for horizontal translation). Forward (backward) direction of translation is abbreviated "f" ("b") and high (low) elevation is abbreviated "H" ("L"). Thus, the term "b, L" ("f, H") refers to the backward (forward) translation of a 2°x1.5° square at -29.5° (+25.5°) elevation. A, B. In early screening experiments, short sequences such as b,H—f,H (A) and its control condition f,H—b,H (B) revealed rapidly adapting, directionally-unselective neuronal responses. C, C'. Subsequently, longer sequences such as 3x b,H—f,H—b,H (C) or 3x b,H—b,L—b,H (C') were used to further investigate the course and determinants of the adaptation. Intervals between the individual stimuli were about 1.3 s in duration for the short sequences (A, B) and about 500 ms for the longer ones (C, C').
(EPS)

S2 Fig Adaptation to moving small-field stimuli irrespective of changes in movement direction. The scatter plots (bubble plots) differentiate the pooled data distributions shown in Fig 4A with respect to the occurrence of changes in movement direction. Trends from a more pronounced response (left respective box plot) towards zero change in spike rate (right respective box plot) suggest that the rapid adaptation observed across the first two stimulus presentations was independent from changes of the direction of motion (through an unchanged region in the visual field).
(EPS)

S1 Table Raw data underlying plots and statistics in Figs 4 and 6. (XLS)
